# Triploid atlantic salmon (*Salmo salar L*.) post-smolts accumulate prevalence more slowly than diploid salmon following bath challenge with salmonid alphavirus subtype 3

**DOI:** 10.1371/journal.pone.0175468

**Published:** 2017-04-12

**Authors:** Lindsey J. Moore, Tom Ole Nilsen, Jiraporn Jarungsriapisit, Per Gunnar Fjelldal, Sigurd O. Stefansson, Geir Lasse Taranger, Sonal Patel

**Affiliations:** 1 Institute of Marine Research, Bergen, Norway; 2 Uni Research Environment, Bergen, Norway; 3 Department of Biology, University of Bergen, Bergen, Norway; 4 Institute of Marine Research, Matre, Norway; Friedrich-Loeffler-Institut Bundesforschungsinstitut fur Tiergesundheit, GERMANY

## Abstract

Triploid Atlantic salmon *(Salmo salar L*.*)* may play an important role in the sustainable expansion of the Norwegian aquaculture industry. Therefore, the susceptibility of triploid salmon to common infections such as salmonid alphavirus (SAV), the causative agent of pancreas disease (PD), requires investigation. In this study, shortly after seawater transfer, diploid and triploid post-smolts were exposed to SAV type 3 (SAV3) using a bath challenge model where the infectious dose was 48 TCID_50_ ml^-1^ of tank water. Copy number analysis of SAV3 RNA in heart tissue showed that there was no difference in viral loads between the diploids and triploids. Prevalence reached 100% by the end of the 35-day experimental period in both infected groups. However, prevalence accumulated more slowly in the triploid group reaching 19% and 56% at 14 and 21 days post exposure (dpe) respectively. Whereas prevalence in the diploid group was 82% and 100% at the same time points indicating some differences between diploid and triploid fish. Both heart and pancreas from infected groups at 14 dpe showed typical histopathological changes associated with pancreas disease. Observation of this slower accumulation of prevalence following a natural infection route was possible due to the early sampling points and the exposure to a relatively low dose of virus. The triploid salmon in this study were not more susceptible to SAV3 than diploid salmon indicating that they could be used commercially to reduce the environmental impact of escaped farmed fish interbreeding with wild salmon. This is important information regarding the future use of triploid fish in large scale aquaculture where SAV3 is a financial threat to increased production.

## Introduction

Sustainable growth of the aquaculture industry in Norway and indeed globally is dependent on solving both disease and environmental issues. In the aquaculture of Atlantic salmon in Norway, major concerns are an increasing environmental footprint and challenges associated with salmon lice infestations and many viral diseases.

One of the environmental concerns is the Atlantic salmon escaping from marine net pens and interbreeding with wild salmon populations and it has been reported that the degree of introgression is largely dependent on the density of aquaculture sites nearby wild salmon rivers [1]. The use of sterile, triploid Atlantic salmon [2] in culture would avoid farmed, escaped salmon from reproducing in natural wild salmonid habitats. Triploid salmon, although phenotypically indistinguishable from diploids, have shown physiological differences requiring optimization of rearing conditions compared to diploids. This has manifested itself as a relative intolerance to warmer seawater temperatures compared to diploid salmon [3, 4]; the bulk production of triploid salmon takes place in the cooler northern Norwegian waters. Another challenge when rearing triploid salmon has been the optimization of feed composition to overcome skeletal deformities and cataracts that were more common in triploid salmon [5, 6].

Another concern in salmon aquaculture is viral diseases. One of the viral agents, salmonid alphavirus (SAV) causes pancreas disease (PD) in farmed salmonids in northern Europe during the seawater phase causing financial losses from both mortality and reduced filet quality [7]. SAV infection causes inflammation and cellular degeneration in target organs, beginning in exocrine pancreas and followed by heart and then skeletal muscle. PD is a debilitating disease causing fish to lose appetite and although many fish recover they fail to thrive and achieve good market weight. Mortality associated with PD outbreaks can vary between 5 and 90% due to several factors such as virulence of SAV subtype and isolate, husbandry, handling stress and may also depend on the robustness of the fish [8–12].

The susceptibility to diseases commonly encountered in aquaculture needs to be thoroughly evaluated to assess the suitability of triploid fish for commercial use. Comparison of diploid and triploid salmon has been carried out with regard to salmon lice, *Renibacterium salmoninarum* infection and *Aeromonas salmonicida* challenge following vaccination [13–15]. Thus, the susceptibility of triploid salmon to SAV3, and the subsequent development of PD in triploid fish is important information for the future development of the aquaculture industry. In order to mimic a natural route of infection, an established bath challenge model for use with Atlantic salmon post-smolts [16] was chosen for this study. It has been shown that the time taken from exposure to reach a prevalence of 100% within a population is dependent upon the exposure dose [17]. Therefore, diploid and triploid Atlantic salmon post-smolts were bath-challenged with SAV3 to evaluate their susceptibility to infection with a relatively low dose. The strength of this study is the use of a bath challenge model, early sampling time-points and a relatively low dose of virus that was instrumental in unravelling the observed differences in susceptibility to SAV3 in diploid and triploid salmon.

## Materials and methods

### Fish

AquaGen AS supplied diploid and triploid eyed eggs. To achieve triploidisation, immediately after fertilisation, the eggs were split into two equal batches, one batch for diploid and the other for the production of triploids. Triploidisation was performed according to Johnstone and Stet [18]. Briefly, shortly after fertilization the eggs were treated with a hydrostatic pressure shock of 655 bar, which prevents the expulsion of the second polar body and creates triploid embryos of both sexes. In March 2015, Atlantic salmon eyed eggs both diploid and triploid (strain, AquaGen® Atlantic QTL-innOva® IPN/PD) were purchased from AquaGen AS and transferred to Matre Research Station, Institute of Marine Research. The eyed eggs were incubated at 6°C, until hatching. The diploid and triploid fry were kept in separate tanks. The salmon fry were first fed with commercial feed, with no extra nutrient additions for triploids (Skretting AS). The photoperiod was kept at L:D 24:0 until November 2015, when it was changed to L:D 12:12. On the 15th January 2016, the photoperiod was switched back to L:D 24:0 to induce parr-smolt transformation which prepares smolts for life in seawater. In mid-February 2016, diploid and triploid Atlantic salmon smolts were transferred in fresh water to the experimental facilities at the Industrial and Aquatic Laboratory (ILAB), Bergen, Norway. They were kept at 12°C, fed according to appetite with commercial feed, containing no extra nutrient additions for triploids (Skretting AS), and starved for 24 hours before sampling and handling. Fish were anaesthetized using 10 mg/L Metomidate and 60 mg/L Benzocaine before handling and euthanized with 10 mg/L Metomidate and 160 mg/L of Benzocaine before sampling. On the day the experiment started the water temperature was increased to 14°C and maintained at this temperature for the duration of the experiment.

### Virus

SAV3 strain H10 [19] was grown in CHH1 cells in L-15 medium supplemented with 2% FCS (foetal calf serum) and gentamycin (500μg/ml) at 15°C. The amount of infectious SAV3 in the cell supernatant was 5 x 10^6^ ml^-1^ TCID_50_ quantified by end-point dilution.

### Experimental procedures

**Fig 1:**
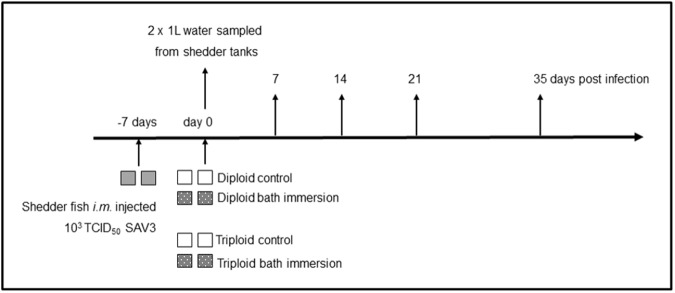
Experimental set up. The time-line line for the experiment showing all tanks and sampling time-points. At the start of the experiment, the average weight of the fish (random 10 fish within each group) was 76.4 ±12.2 g for diploids and 71.6 ±19 g for triploids.

Diploid and triploid smolts that were experimental fish were transferred to seawater (30 ‰) 4 days prior to the start of the experiment and maintained at 12°C. One hundred and thirty additional diploid salmon used as shedder fish were treated similarly. Diploid shedder fish were intramuscularly (*i*.*m*.) injected 1 week prior to the experimental bath challenge (-7 days) with 1.3 x 10^3^ TCID_50_ SAV3 diluted in L-15 cell culture medium and kept in two 150 L tanks (Fig 1). The virus shed by these fish constituted the SAV3 used for the subsequent challenge of diploid and triploid fish. The day the experiment started the water flow was stopped for one hour in the shedder tanks and the shedder fish were removed and euthanized. The tank water from the shedder tanks was pooled and diluted, 1:4. One hundred liters of this diluted seawater, containing infectious virus (shed by the shedder fish) was added into each of the four 150 L tanks where the fish were exposed to SAV3. Two of these four tanks containing SAV3 were populated with 55 diploid fish per tank and the other two tanks with 55 triploid fish per tank. Four control tanks containing the same volume of water (100 L) were populated similarly with 55 diploid or triploid fish to act as non-infected control groups. The exposure to SAV3 was carried out for 6 hours, before the flow was re-started in all tanks according to Jarungsriapisit *et al*. [16]. The design of this experiment was approved by the Norwegian Animal Research Authority (ID: 8413).

### Sampling

Seawater (2 x 1 L) was sampled from each shedder tank, the day the experiment started after the flow had been stopped for one hour (Fig 1). Additional water samples were taken from the four tanks of diluted shedder water. Water samples were vacuum filtered through electropositive Zeta Plus^®^ Virosorb^®^ 1 MDS filters (Cuno Inc., USA) using Millipore^®^ sintered glass filters and funnels [20]. The virus was eluted from these filters in 1.2 mLs of L-15 medium containing 10% FCS, thereby concentrating a litre of tank water into 1.2 mL according to Jarungsriapisit *et al*. [17]. The virus in the eluant was quantified by measuring SAV3 RNA by qPCR (see below) and by TCID_50_ end-point titration in order to determine the bath immersion dose. Eight post-smolts were sampled from each tank at 7, 14, 21 and 35 days post exposure (dpe), and their weights and fork-lengths recorded for condition factor calculation using the following formula:

$$CF=\left[fish\;weight\;\left(g\right)\;x100\right]/{\left[fish\;length\;\left(\text{-}cm\right)\right]}^{3}$$

Heart samples from all sampled fish were flash frozen in liquid nitrogen and stored at -80°C until analysis. Heart and pancreas tissues were sampled for histology from the same 8 fish at 14 dpe when typical PD pathology is most commonly observed in both heart and pancreas and fixed in 10% neutral buffered formalin. Adipose fins were collected and stored in ethanol from all sampled triploid fish to verify triploidy.

### Total RNA preparation

Heart tissue samples (half the heart) were homogenized in 1 ml TRIzol® using a Precellys 24 instrument and 1.4 mm diameter Zirconium oxide beads. After the homogenization and chloroform treatment, 450 μl of supernatants were used further for RNA isolation using a Purelink total RNA extraction kit, in an iPrep machine (Life Technologies). RNA from filtered/concentrated seawater eluants was isolated by mixing 100 μl eluant with 350 μl lysis buffer, and further isolation was carried out by Purelink total RNA extraction kit (Life Technologies). RNA was eluted in 50 μl of the propriety buffer. RNA concentration and quality was estimated using a Nanodrop 1000 ND. Ten percent of the samples were checked for integrity on a Bioanalyser (Agilent Instruments) resulting in RINs ≥ 8.

### cDNA and RT-qPCR for viral copy number quantification

SAV3 RNA from heart tissue was quantified with a modified one-step nsP-1 assay (Ag-Path, Ambion) [21] with a sense probe, using 200 ng total RNA in a total reaction volume of 10 μl. Approximately twenty percent of heart RNA samples randomly selected from all groups and time-points were qualitatively verified by measuring the transcription of elongation factor 1A [22]

SAV3 RNA isolated from filtered/concentrated water samples was quantified using a two-step assay and together with the TCID_50_ result constituted the virus dose the fish were exposed to. cDNA was transcribed from 2 μl total RNA in a 10 μl reaction using SuperScript™ VILO™ (Invitrogen) as described in the manufacturer’s instructions. qPCR was run in triplicate in 96 well plates using TaqMan® Fast Universal Master Mix (Applied Biosystems®) and an Applied Biosystems 7900H Fast sequence detection analyser. A 10 μl reaction volume contained; 2 μl cDNA (diluted 1:10), 5 μl 2 x master mix, 900nM of each primer and 250nM of FAM-labelled probe. The running conditions were as recommended by the manufacturer.

SAV3 RNA was quantified using a standard curve produced using a 10 x dilution series of synthetic SAV3 RNA (576 bps, cRNA) containing 10^1^ to 10^7^ copies of SAV RNA [17]. These standards were analysed on each plate when quantifying copy numbers from heart RNA in the one-step assay. The same dilutions were reverse transcribed (as above) and run in duplicate for the quantification of copy numbers in the two-step assay for water samples.

### Histology

Formalin fixed tissues were paraffin embedded and 3μm sections Haematoxylin-Erythrosin-Saffron (HES) stained before visualizing under a Leica DMRBE light microscope (Leica Microsystems, Germany). Photographs were taken using Spotflex camera model nr 15.2 64 Mp Shifting pixel (Diagnostic instruments Inc, USA) and processed with Image J software.

### ATPase analysis

Smolt status was analysed by measurement of Na^+^/K^+^ ATPase activity (NKA) in gill tissue 2 weeks before the start of the experiment and before the fish were transferred to the experimental facility. Analysis was carried out as described previously [23] and results expressed as μmol ADP mg protein^−1^ h^−1^.

### Triploid determination

Before transfer to the experimental facility ploidy was verified by measuring erythrocyte diameter on blood smears on 10 triploids [24]. For fish sampled during the experiment genomic DNA was extracted (Qiagen, DNeasy) from adipose fins collected from all triploid fish. Triploid status was verified using multi-loci microsatellite genetic profiling; the presence of three alleles at multiple loci [25], and any fish not triploid at all loci investigated were excluded from further analysis.

### Data analysis

SDS software version 2.4.1 automatically calculated copy numbers of the nsP-1 transcript from the Ct values of a standard curve run on the same plate. These copy numbers were analysed using Statistica version 12.7. Condition factors between infected and control fish within diploid and triploid groups at all time-points were compared using a t test (MS Excel 2016).

## Results

### Fish

**Fig 2:**
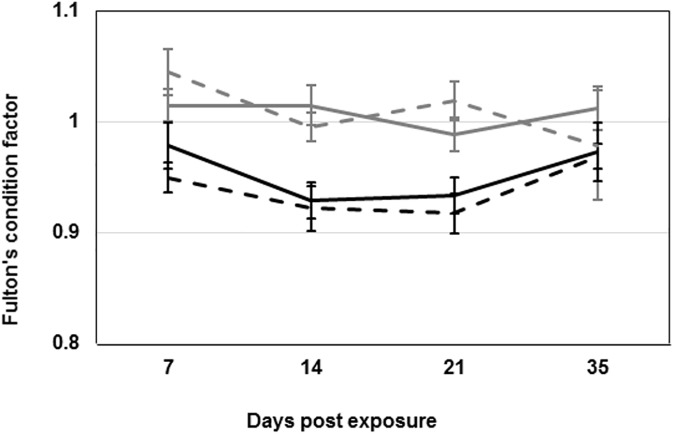
Condition factor. Fulton’s condition factor calculated by: 100 × weight (g)/ [fork length (cm)]^3^ for all fish sampled at all time-points. Lines represent the average ± SEM per group. Black lines represent triploid groups and grey lines the diploid groups. Dashed lines represent control groups.

All fish within the triploid group were triploid when tested before transfer to ILAB (erythrocyte size on blood smears). Of the 74 experimental fish tested (multiple loci microsatellite mapping) one was diploid and one sample was contaminated and these 2 fish were excluded from further analysis. Condition factors calculated from weights and lengths of all fish remained stable and were not significantly different within the diploid and triploid salmon when compared to their respective non-infected controls throughout the experiment (Fig 2).

### Bath immersion dose

On the day of exposure, water from the shedder tanks contained an average of 160 copies of SAV3 RNA per litre and the TCID_50_ assay revealed 48 TCID_50_ SAV3 per litre. The shedder water was diluted 1:4 and used for exposure to potentially infect the diploid and triploid salmon. Water sampled after the 1:4 dilution produced unreliable results in the copy number analysis and, therefore, the result for the undiluted water was used and divided by 4 (the dilution factor).

### PD status

**Fig 3:**
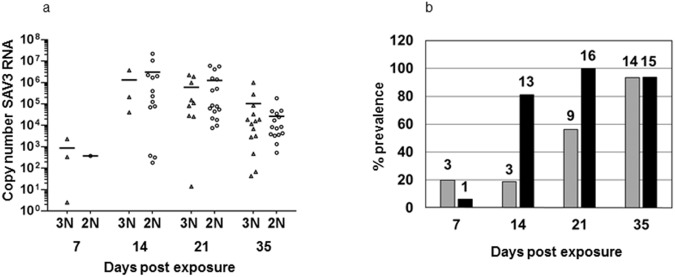
SAV3 Status. **3a**. Copy numbers of SAV3 RNA (viral load) of all individuals in triploid (Δ, 3N) and diploid (O, 2N) groups at all time-points. **3b**. Percentage positive fish for SAV3 RNA in the triploid group (grey bars) and diploid group (black bars) at all time-points. Numbers above the columns are the number of positive fish (n = 16 except in the triploid group at 7 and 35 dpe where n = 15).

SAV3 status was determined by copy number analysis of SAV3 RNA in total RNA from heart tissue (Fig 3A). Copy number analysis showed a 100% prevalence in the diploid group by 21 dpe, whereas the prevalence in the triploid group rose more slowly reaching 93.3% by the end of the experiment (35 dpe) (Fig 3B). Although slower to reach a maximum prevalence the triploid group showed similar viral loads (copy numbers of SAV3 RNA) to the diploid group at all time-points (Fig 3A). There was no significant difference in the viral loads between the diploid and the triploid groups (p ≥ 0.05) at any of the time-points.

**Fig 4:**
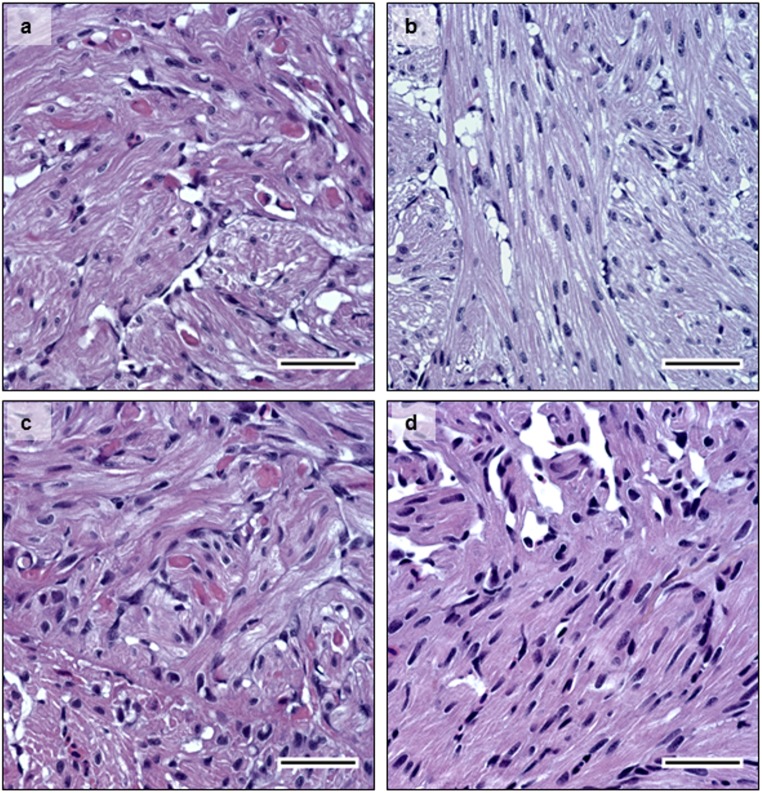
Histology. HES staining of pancreas tissue sections at 14 dpe. **4a-** infected diploid, **4c-** infected triploid, showing cell degeneration. **4c-** non-infected diploid; and **4d-** non-infected triploid. Infected fish showed loss of exocrine pancreatic cells and immune cell infiltration, while non-infected controls show normal histology in pancreas. Bar = 50μm.

**Fig 5:**
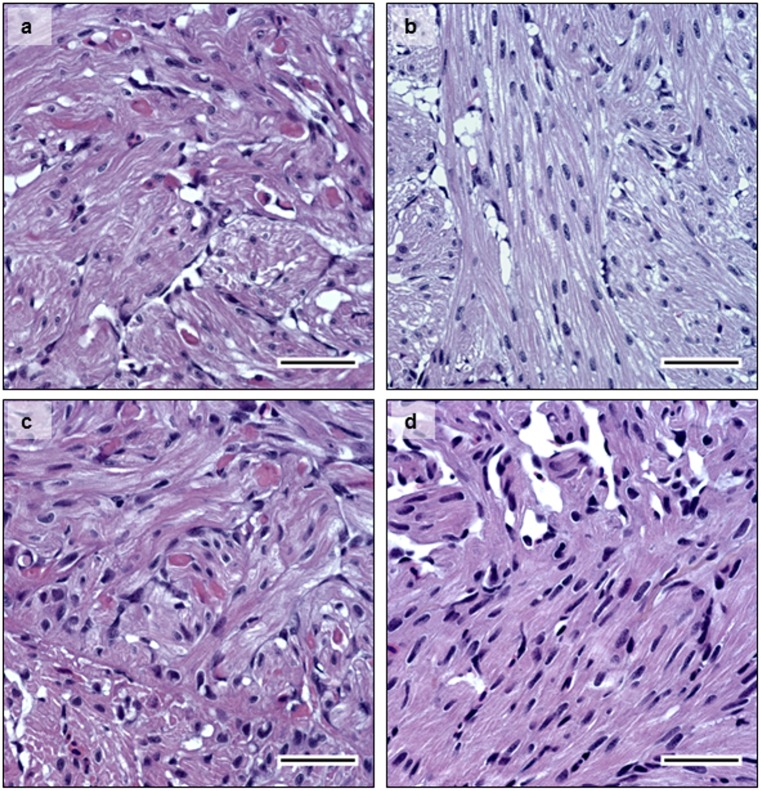
Histology. HES staining of heart tissue sections at 14 dpe. **5a-** infected diploid, **5b-** infected triploid, showing myocardial cell degeneration. **5c-** non-infected diploid; and **5d-** non-infected triploid, show normal histology in heart tissue sections. Bar = 50μm.

Additionally, PD status was confirmed histologically at 14 dpe when pathology is usually observed in both heart and pancreas. PD-associated lesions such as degeneration of exocrine pancreatic cells (Fig 4A–diploid, and 4B–triploid), and degeneration of myocardial cells in the heart were observed (Fig 5A–diploid, and 5B—triploid). Loss of exocrine pancreatic cells and infiltration by inflammatory cells was observed in pancreas tissue at 14 dpe in both diploid and triploid fish (Fig 4A and 4B respectively). The degree of histopathological changes in the infected individuals was independent of whether the fish were diploid or triploid. None of the control fish examined showed any PD-associated histopathological changes in the pancreas (Fig 4C and 4D), or in the heart (Fig 5C and 5D).

### Na^+^/K^+^ ATPase status

High gill NKA activities in all diploid and triploid fish tested before the start of the experiment (n = 10) showed that these groups of fish were ready for seawater transfer. Diploid fish had an average of 14.6 μmol ADP mg protein^−1^ h^−1^ (range: 11.0 to 20.0) and triploid fish an average of 16.3 μmol ADP mg protein^−1^ h^−1^ (range: 12.3 to 21.7).

## Discussion

At 35 dpe 93% of both the diploid and the triploid SAV3 exposed fish were positive for SAV3 RNA. In addition, histological examination showed the development of PD-associated lesions in these groups. Prior to seawater transfer diploid and triploid smolts showed gill NKA activity commensurate with good quality smolts and good seawater tolerance. The aquaculture industry aims to transfer only good quality smolts to sea cages so that the fish will be robust and capable of fighting pathogens. However, it has been reported that even though salmon smolts are within the smolt window when transferred to seawater they are more susceptible to SAV3 during the first few weeks [16]. In marine sea cages, the dose of viral agents that salmon would be exposed to is anticipated to be very low. By choosing both a relatively low dose and smolts recently transferred to seawater this experiment was sufficiently sensitive to ensure that at least a few individuals would be infected, and the difference in the accumulation of prevalence between diploid and triploid salmon could be observed. Since both diploid and triploid salmon in this study were raised from the same batch of eggs, had comparable NKA activities before seawater transfer, and were exposed to virus simultaneously the differences in their accumulation of prevalence to SAV3 could be attributed to their difference in ploidy. The condition factor was not affected by SAV3 infection in the five weeks experiment in this study. However, a reduction in condition factor has been reported in a similar experiment with SAV3 and Atlantic salmon post-smolts during a longer 12 weeks experiment [9].

The exposure to SAV3 was estimated by both nsP-1 (SAV3 RNA) copy number analysis and by end-point dilution of infectious virus (TCID_50_ L^-1^ in the tank water). Although the numbers of virus per litre measured by TCID_50_ is considerably less than that measured by the copy number analysis, it represents a live infectious dose and not SAV3 RNA, and thus the results from the two methods are not directly comparable. Since prevalence approached 100% in all infected tanks, the exposure dose was clearly sufficient to cause infection.

Previous studies have failed to demonstrate major differences in disease resistance between diploid and triploid Atlantic salmon. An earlier study involving all female diploid and triploid groups of salmon was unable to find any differences in the susceptibility to bacterial kidney disease due to ploidy differences [13]. Triploid Chinook salmon *(Oncorhynchus tshawytscha)* showed an increased mortality following *Vibrio anguillarum* challenge, but only under stressful conditions [14]. Diploid and triploid siblings performed equally well when vaccinated and then challenged with *Aeromonas salmonicida* although there were differences in their white cell numbers and respiratory burst activity [15]. Finally, the susceptibility to sea lice infestation in triploid Atlantic salmon, investigated at different life stages both in Scotland and in Norway, concluded that triploid salmon showed no increased susceptibility to lice infestation compared to diploids [26].

The fish used in this study were from the same batch of AquaGen® QTLinnOva ® IPN/PD strain that possess an increased resistance to viral diseases. They were infected using an experimental model that attempts to mimic the conditions at aquaculture sites, but both the exposure time and dose were likely higher than experienced during a field infection, thus 100% prevalence was attained rather rapidly compared to a field outbreak where prevalence increases more gradually within a sea-cage.

These results are from an experimental infection using relatively small groups of fish and a single isolate of SAV3. It could be of interest to investigate the effects of varying parameters such as other virus isolates or sub-types, size and physiological status of fish on the susceptibility of SAV3. Differences in mortality between isolates has been documented [9]. The temperature of 14°C used in this study is an optimum temperature for diploid salmon post-smolts [27, 28] of the weight used in this experiment, while triploid post-smolts have a lower temperature optimum [29]. In addition, all the fish in this study received standard feed for diploid fish although special nutrient formulations specifically for triploid fish to optimize their growth rates and reduce skeletal deformities have been recommended. However, sub-optimal temperature and sub-optimal feed had no detrimental effect on the maximal prevalence in the triploid group compared to the diploid group, and the prevalence accumulated more slowly in the triploid group.

It is not known why triploids during this experiment exhibited a slower accumulation of prevalence, but differences in the proportions of immune cells in the head kidney have been reported between triploid and diploid Atlantic salmon following vaccination [15, 30]. These differences may however be compensating for the slightly larger cell size in triploid fish [31, 32], but could also indicate differences in the immune response in these fish groups. In conclusion, although no significant difference in the prevalence of SAV3 could be seen between triploids and diploids at the end of a 5 weeks experimental period, the virus appeared to spread more slowly through the triploid fish group. Under field conditions where even lower doses are likely this slower accumulation of prevalence could either delay the onset of PD or avoid an outbreak altogether. Such a scenario would be a significant advantage in the months before harvest when mortality from PD is financially the most damaging. Further investigation of possible differences when triploids and diploids are exposed to very low amounts of virus, more closely mimicking field conditions, would be interesting. The triploid group in this study showed no increased susceptibility or pathology when exposed to SAV3 virus indicating that their commercial use could reduce the environmental impact of escaped farmed fish interbreeding with wild salmon.

## Supporting information

S1 Raw data (XLSX)

## Abbreviations

ADP: adenine diphosphate
CHH1: Chum salmon heart cells 1
cRNA: synthetic RNA
dpe: days post exposure
FCS: foetal calf serum
NKA: sodium /potassium ATPase activity
L:D: light:dark
PD: pancreas disease
SAV3: salmonid alphavirus type 3
TCID_50_: 50% tissue culture infectious dose
