# Correction: Triploid atlantic salmon (*Salmo salar L*.) post-smolts accumulate prevalence more slowly than diploid salmon following bath challenge with salmonid alphavirus subtype 3

**DOI:** 10.1371/journal.pone.0177250

**Published:** 2017-05-03

**Authors:** Lindsey J. Moore, Tom Ole Nilsen, Jiraporn Jarungsriapisit, Per Gunnar Fjelldal, Sigurd O. Stefansson, Geir Lasse Taranger, Sonal Patel

Fig 4 is a duplicate of Fig 5. Please see the correct Fig 4 and its caption below.

**Fig 4:**
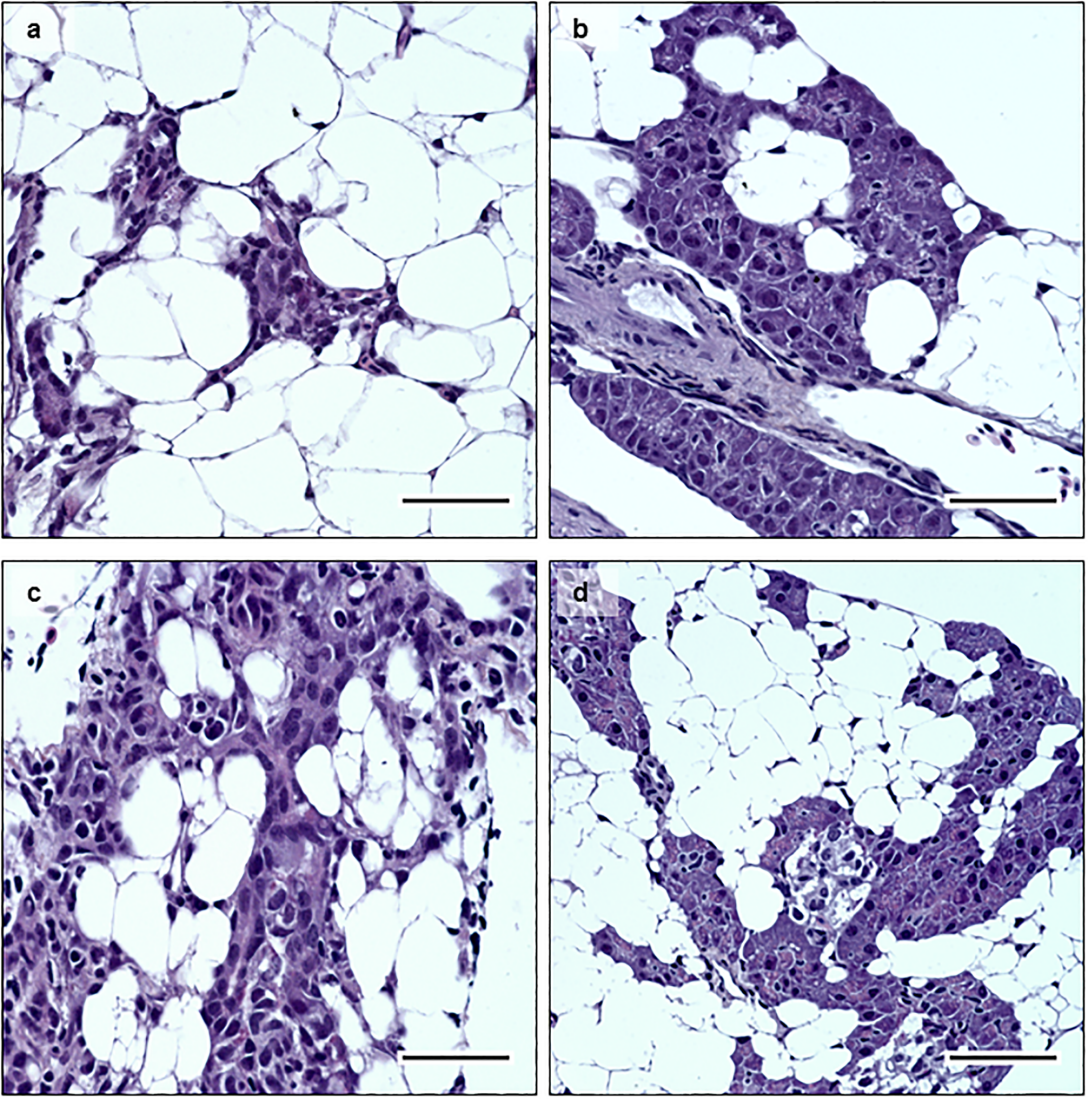
Histology. HES staining of pancreas tissue sections at 14 dpe. 4a- infected diploid, 4c- infected triploid, showing cell degeneration. 4c- non-infected diploid; and 4d- non-infected triploid. Infected fish showed loss of exocrine pancreatic cells and immune cell infiltration, while non-infected controls show normal histology in pancreas. Bar = 50μm.
